# Doublesex and GATAβ4 synergistically regulate the sex-dimorphic expression of storage protein 1 in *Bombyx mori*

**DOI:** 10.1371/journal.pgen.1011762

**Published:** 2025-07-11

**Authors:** Jiamin Yan, Haonan Dong, Tingting Tian, Chunxia Xiao, Yuanyuan Sun, Jing Gong, Qingyou Xia, Yong Hou

**Affiliations:** 1 Integrative Science Center of Germplasm Creation in Western China (CHONGQING) Science City, Biological Science Research Center, Southwest University, Chongqing, China; 2 College of Sericulture, Textile and Biomass Sciences, Southwest University, Chongqing, China; 3 Key Laboratory for Germplasm Creation in Upper Reaches of the Yangtze River, Ministry of Agriculture and Rural Affairs, Chongqing, China; Peking University, CHINA

## Abstract

Sexually dimorphic traits are widespread in organisms and are crucial for reproduction and behavior. These traits are typically controlled by sex-specific genes. However, their regulatory mechanisms are complex and incompletely understood. In *Bombyx mori*, a group of sex-differential storage proteins (SPs) exists, with storage protein 1 (SP1) expressed exclusively in females. In this study, we used the CRISPR/Cas9 system to knock out the *doublesex* gene and found that SP1 expression was sharply upregulated in male doublesex mutants and downregulated in female doublesex mutants, which suggests that doublesex is a key factor in the sex-differential expression of SP1. Then, we revealed that the female-specific doublesex isoform (dsxF) bound to and activated the *SP1* promoter more strongly than the male-specific isoform (dsxM). Meanwhile, a transcription factor named GATAβ4 was found to be involved in the regulation by doublesex. Overexpression of GATAβ4 in *Bombyx mori* larvae affected adult reproductive behavior and dramatically upregulated SP1 expression in males. Furthermore, GATAβ4 interacted with both dsxF and dsxM, promoting nuclear translocation of dsxM, which in turn inhibited GATAβ4 binding to the *SP1* promoter. In total, we found that dsxM did not directly repress SP1 expression in males but instead cooperated with other transcription factors to regulate downstream gene expression. These findings provide new insights into the regulation of sex-specific genes and the mechanisms controlling dimorphic traits.

## Author summary

Sex-specific expressed genes are widely present across various organisms and play a crucial role in reproduction and evolution. However, the regulatory mechanisms governing their expression remain relatively poorly understood. In this study, we used the female-specific expression gene *SP1* in the silkworm as a model and demonstrated that mutation of the sex-determining transcription factor doublesex or transgenic overexpression of the nutritional transcription factor GATAβ4 successfully induced high SP1 expression in male silkworms. Our findings indicate that in female silkworms, both dsxF and GATAβ4 directly regulate the high expression of SP1. In contrast, in male silkworms, the direct regulatory effect of dsxM on SP1 is weak. Instead, dsxM primarily interacts with GATAβ4, inhibiting its binding to the *SP1* promoter and thereby blocking GATAβ4-mediated SP1 upregulation, ultimately leading to low SP1 expression. These findings suggest a potential mechanism regulating sex-specific gene expression.

## Introduction

Sexual dimorphism refers to the significant differences in morphology, behavior, and physiological characteristics between males and females of the same species, resulting from the combined effects of sexual selection and natural selection [1]. This phenomenon is widespread in the animal kingdom, manifesting in various forms such as body size, coloration, ornamental traits, vocalization, and behavior [2]. Modern research employed methods from genetics, behavior, and ecology to explore the mechanisms of sexual dimorphism and its crucial role in evolution [3].

Biological dimorphism reflects a range of potential molecular mechanisms within systems, with sex specificity in mammals determined by gonadal hormones [4]. Subsequent research revealed that a large family of similar transcription factors is involved in sex determination. The male sex regulatory gene mab-3 was isolated in *Caenorhabditis elegans* and the sex regulatory gene doublesex was identified in *Drosophila melanogaster*, both of which contain a DM domain [5]. In vertebrates, a gene containing the DM domain called Dmrt1 has been found to regulate male development [6]. Mab-3 and Dmrt1 both influence male sexual development through sex-specific expression; mab-3 controls the male tail tip morphology in *Caenorhabditis elegans* [7], and Dmrt1 regulates the dimorphism of internal and external genitalia in *Mus musculus* [6,8].

In insect sex cascade control, certain genes ultimately regulate the sex-specific splicing of doublesex, resulting in the production of sex-specific doublesex proteins in females and males [9–13]. In the red flour beetle, *Tribolium castaneum*, doublesex plays a direct role in regulating female fertility by controlling the expression of vitellogenin, its receptor, and other reproduction-associated genes [12]. In social insect termites, doublesex is proposed to sustain reproductive division of labor by modulating genes involved in spermatogenesis and lifespan regulation, including piwi-like proteins [14]. In *Papilio polytes* butterflies, doublesex exerts dual regulatory effects: it activates Wnt signaling pathway genes to promote mimetic wing pattern formation while repressing genes such as abdominal-A to suppress non-mimetic traits [15]. The formation of male-specific abdominal pigmentation in *Drosophila melanogaster* is controlled by doublesex and Abdominal-B (Abd-B) [16,17]. Doublesex and Abd-B jointly regulate the number of abdominal segments in male and female *Drosophila melanogaster* [18]. Doublesex and *sex combs reduced* (Scr) cyclically regulate the appearance of sex combs of male *Drosophila melanogaster* [19,20]. Doublesex and fruitless (fru) play a crucial role in the normal development of the nervous system and the normal occurrence of sexual behavior in male and female *Drosophila melanogaster* [21,22]. Additionally, doublesex regulates the courtship behavior of *Drosophila melanogaster* [23]. Genome-wide analyses in *Drosophila* have revealed that although both dsxM and dsxF can bind to the genomic regions, their targets include transcription factors and components of signaling pathways that directly and indirectly regulate sexually dimorphic gene expression [24].

Sex-specific splicing of doublesex (dsxF and dsxM) regulates target genes, notably demonstrating established regulation of the yolk protein (yp) in *Drosophila melanogaster*. The promoters of the *yp1* and *yp2* in *Drosophila melanogaster* contain a fat body-specific enhancer element known as FBE [25,26]. DsxF and dsxM directly bind to several specific sites on FBE both *in vitro* and *in vivo* to regulate the expression of the yolk proteins [27,28]. The FBE enhancer comprises the dsxA and bzipl elements. Using site-specific mutations, the authors determined the functional roles of these elements within FBE and discovered that in the female fat body, the expression of yolk protein is synergistically activated only when both dsxA and bzipl elements are present. However, in the male fat body, this simultaneous presence did not lead to expression. It is the female-specific dsxF protein, rather than the male-specific dsxM protein, that collaborates with the protein bound to the bzipl element to activate transcription [9,29,30]. However, the effects of dsxF and dsxM on the binding of other transcriptional activators are not completely understood, nor is the identity of these transcriptional activators. Therefore, the mechanisms by which doublesex regulates gene expression patterns and controls male-specific or female-specific morphological traits in insects are still not well understood.

In the Lepidoptera model organism, the silkworm *Bombyx mori*, doublesex undergoes sex-specific splicing to produce the isoforms dsxF and dsxM [31]. Using transcription activator-like effector nuclease (TALEN) technology to specifically mutate the female-specific exon of the doublesex in the silkworm results in female mutants lacking egg storage and exhibiting abnormal external genitalia, leading to female-specific sterility [32]. Using transgenesis technologies to induce mutations in either the male-specific isoform (dsxM) or the common region (dsxC) of the doublesex in silkworms result in either male-specific sexual dimorphism defects or intersex phenotypes [33]. The pheromone-binding protein (PBP) [34,35], vitellogenin [36–38], and storage protein 1 [39] in *Bombyx mori* are all sex-specific expressed genes, providing an ideal system to study the regulatory mechanisms of doublesex on gene expression patterns. Storage protein 1 is a plasma protein known as hexamerin, synthesized exclusively in the fat body of final instar larvae of female silkworms and released into the hemolymph for accumulation [36,40]. The expression of storage protein 1 exhibits clear sex-specificity, with significantly higher levels in females and nearly absent expression in males [39,41]. Knocking out storage protein 1 leads to a drastic reduction in egg hatching rate [42]. However, the mechanisms underlying the sex-specific expression of these genes have not been fully clarified.

In this study, we found that doublesex in the silkworm controlled various dimorphic traits, including internal and external reproductive organs, the number of abdominal segments, and wing pattern coloration in female moths. The high expression of SP1 in females is cooperatively regulated by the sex regulatory gene doublesex and the nutritional transcription factor GATAβ4. These findings provide valuable insights into the mechanisms governing dimorphic traits in insects.

## Results

### Dimorphism abnormality induced by doublesex mutation

**Fig 1:**
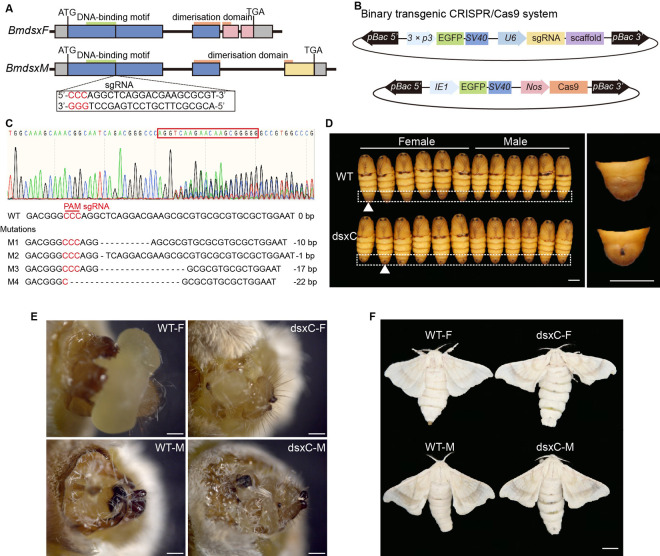
Doublesex mutations induce changes in dimorphism. (A) The *doublesex* gene undergoes alternative splicing to produce female-specific (BmdsxF) and male-specific (BmdsxM) isoforms in *Bombyx mori*. Grey boxes represented the 5’ and 3’ untranslated regions (UTRs); blue boxes represented the doublesex common region; pink boxes indicated female-specific exons; yellow boxes indicated male-specific exons; green rectangle indicated the DNA-binding motif; orange rectangle indicated the dimerization domain. ATG denoted the start codon, TGA denoted the stop codon, and numerals indicated the number of nucleotides. The sgRNA represented the CRISPR/Cas9 site and the sequence shown in the dotted box. (B) The binary transgenic CRISPR/Cas9 system consists of two plasmids: one containing the sgRNA driven by the *U6* promoter and the EGFP reporter gene driven by the *3 × P3* promoter, and the other containing the Cas9 and EGFP genes controlled by the *Nanos* promoter and the *IE1* promoter, respectively. SV40 represents a transcriptional terminator. (C) Sequencing results and mutation types in F1 progeny after hybridization of Cas9 and sgRNA. The red box showed the sgRNA sequence, with the PAM sequence in red. WT denoted wild type, and M1 ~ M4 indicated different mutant types. Missing bases are represented by dashes, and the number of missing bases is shown to the right of the sequence. (D) Comparison of pupa abdomens between wild type and dsxC mutants. The dashed white frame indicated the last body segment of the pupa. The white triangle pointed to the pupa magnified on the right. WT denoted wild type, and dsxC denoted the doublesex common region mutant. Female and Male indicated the sex of the pupa. (E) Morphology of external genitalia in wild type and dsxC mutant moths. (F) Comparison of wing color and the number of body segments in wild type and dsxC mutant moths. WT-F: wild type female moths; WT-M: wild type male moths; dsxC-F: dsx common region mutant female moths; dsxC-M: doublesex common region mutant male moths. Scale bar, 500 μm.

The *doublesex* gene is a candidate regulator of sex-specific gene expression. To test whether doublesex influences sex-specific gene expression, the common region of all splicing isoforms (dsxC) in exon 1 was targeted using single guide RNA (sgRNA) to the CRISPR/Cas9 system (Fig 1A). One vector containing sgRNA and Enhanced Green Fluorescent Protein (EGFP) was driven by *U6* [43] and *3 × P3* promoters [44], respectively. A second vector containing Cas9 and EGFP was controlled by *Nanos* (*nos*) [45] and *immediate-early 1* (*IE1*) promoters [33,46], respectively (Fig 1B). The *U6*-sgRNA parental transgenic line was established (S1A and S1B Fig) and the Cas9 parental transgenic line was reared (S1C Fig). Polymerase Chain Reaction (PCR) and sequence analysis indicated that both F1 progeny male and female individuals had mutations at the sgRNA site, most of which were base deletions (Fig 1C). In the F1 progeny, both male and female moths exhibited abnormal external genitalia (Fig 1E). Female moths developed an eighth ventral segment, while male moths maintained their eight ventral segments (Fig 1F). These results were consistent with the previously reported abnormalities in sexual dimorphism caused by doublesex mutations [32,33]. Additionally, we observed a distinct black spot on the last abdominal segment in both female and male pupae (Fig 1D), as well as a deepening of wing pattern coloration in female moths (Fig 1F).

### Doublesex mutations alter the expression of storage proteins

**Fig 2:**
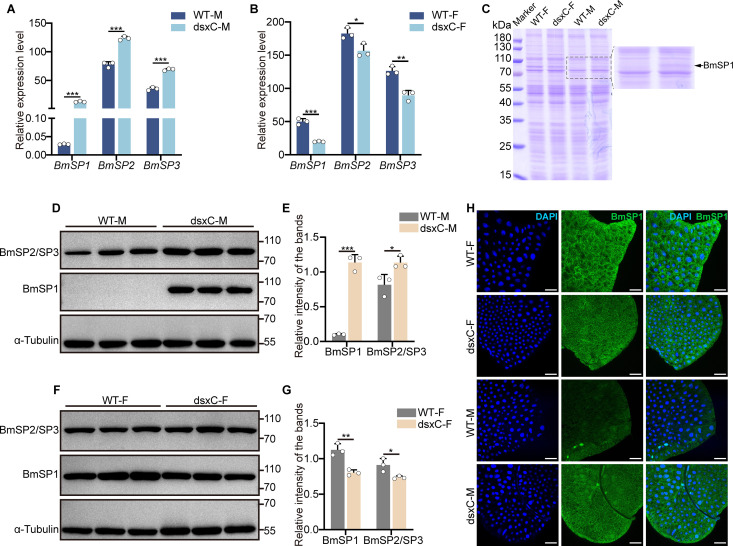
Analysis of storage protein expression after mutation in the doublesex common region. (A) QRT-PCR analysis of *SP* mRNA expression in the fat body of wild type and dsxC mutant male larvae on the 3 rd day of the fifth instar. (B) Expression of *SP* mRNA in the fat body of wild type and dsxC mutant female larvae on the 3 rd day of the fifth instar. (C) SDS-PAGE analysis of total proteins (10 μg/lane) in the fat body of wild type and dsxC mutant larvae on the 3 rd day of the fifth instar. The black dashed box highlights SP expression, with the black arrow pointing to SP1. (D) Western blot analysis of SP protein expression in the fat body of wild type and dsxC mutant male larvae on the 3 rd day of the fifth instar. (F) Expression of SP proteins in the fat body of wild type and dsxC mutant female larvae on the 3 rd day of the fifth instar. (E, G) Statistical analysis of the data in (D) and (F). (H) Immunofluorescence analysis of SP1 protein expression in the fat body of wild type and dsxC mutant larvae on the 3 rd day of the fifth instar. Scale bar, 50 μm. WT-F: wild type female larvae; WT-M: wild type male larvae; dsxC-F: doublesex common region mutant female larvae; dsxC-M: doublesex common region mutant male larvae. All experiments were conducted with three biological replicates, with each group consisting of five silkworms. Error bars represent mean ± SD (*n* = 3). Statistical significance was determined using two-tailed Student’s *t*-tests and is indicated by *P < 0.05, **P < 0.01, and ***P < 0.001.

Storage proteins (SPs) are plasma proteins known as hexamerins, and SP1 is a female-specific protein synthesized by the fat body and accumulated in the hemolymph of larvae [36,40]. Notably, in male dsxC mutants, *SP1* mRNA was extremely upregulated (Fig 2A), which was also evidenced by SDS-PAGE (Fig 2C) and western blot (Fig 2D and 2E) as well as by the immunofluorescence localization in the fat body (Fig 2H). In female dsxC mutants, SP expression was downregulated at both mRNA (Fig 2B) and protein levels (Fig 2F and 2G), but not as low as in wild-type males.

### DsxF and dsxM differentially bind to the promoter of *storage protein 1*

**Fig 3:**
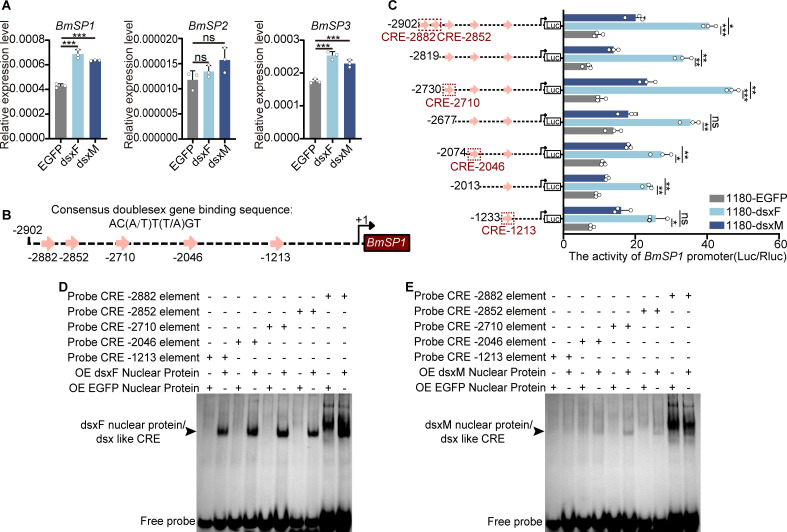
Investigation of direct regulation of storage protein expression by dsxF and dsxM. (A) Expression of *SPs* mRNA in BmE cells 48 hours after overexpression of dsxF, dsxM, or enhanced green fluorescent protein (EGFP). (B) Diagram of the *SP1* upstream promoter and potential doublesex binding sites. “+1” denoted the transcriptional start site (TSS); the black dotted line represented the 2902 bp *SP1* promoter sequence upstream of the TSS in the genome. The pink arrows represented potential doublesex binding sites, with the numbers below indicating the distance between these sites and the TSS. (C) Activity analysis of *SP1* promoters truncated according to the potential doublesex binding sites after overexpression of dsxF, dsxM, or EGFP in BmE cells for 48 hours. (D) Electrophoretic mobility shift assay (EMSA) of five biotin-labeled doublesex cis-regulatory elements (CREs) with 6 μg of nucleoprotein extracted from BmE cells 48 hours after overexpression of dsxF or EGFP. (E) EMSA of five biotin-labeled doublesex CREs with 6 μg of nucleoprotein extracted from BmE cells 48 hours after overexpression of dsxM or EGFP. The biotin-labeled probe concentrations were 50 μM. “+” indicated the presence of the sample in the lane; “-” indicated its absence. The black arrows point to the shifted bands. OE: overexpression. All experiments were conducted with three biological replicates. Error bars represent mean ± SD (*n* = 3). Statistical significance was determined using two-tailed Student’s *t*-tests and is indicated as ns, no significant difference; *P < 0.05, **P < 0.01, and ***P < 0.001.

To investigate the effects of dsxF and dsxM on the expression of SPs, we overexpressed dsxF and dsxM in BmE cells. QRT-PCR analysis revealed that *SP1* and *SP3* mRNA levels were upregulated in both dsxF and dsxM. The degree of upregulation was more pronounced in dsxF compared to dsxM (Fig 3A). The analysis of promoter of *SP1* revealed five potential doublesex binding cis-regulatory elements (CREs) located at -1213 bp, -2046 bp, -2710 bp, -2852 bp, and -2882 bp within the 3000 bp upstream region (Fig 3B). Overexpression of dsxF or dsxM in BmE cells promoted the activity of *SP1* promoter (Fig 3C), consistent with the mRNA results. Electrophoretic mobility shift assays (EMSAs) were performed and the results showed that nuclear proteins overexpressing dsxF strongly bound to CREs at -1213 bp, -2046 bp, -2710 bp, and -2852 bp, with only nonspecific binding at -2882 bp (Fig 3D). By comparison, the nuclear proteins overexpressing dsxM exhibited very weak binding to CREs at -2710 bp and -2852 bp (Fig 3E). It appeared that dsxM did not directly inhibit SP1 expression because of an obvious difference in its DNA-binding affinity compared to dsxF. We hypothesize that dsxM may interact with other transcription factors to exert an inhibitory effect in males.

### Overexpression of GATAβ4 increased storage protein 1 expression in male silkworms

**Fig 4:**
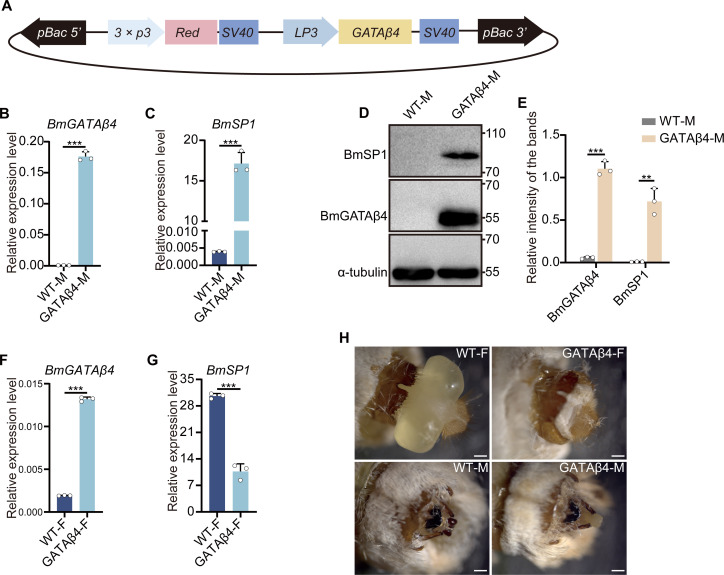
Transgenic overexpression of GATAβ4 in silkworms. (A) Schematic diagram of the transgenic vector containing GATAβ4 driven by the *LP3* promoter and the dsRed reporter gene driven by the *3 × P3* promoter. (B, C) QRT-PCR analysis of *GATAβ4* and *SP1* mRNA in the fat body of wild type and transgenic male larvae on the 3 rd day of the fifth instar. (D) Western blot analysis of GATAβ4 and SP1 proteins in the fat body of wild type and transgenic male larvae on the 3 rd day of the fifth instar. (E) Statistical analysis of the data in (D). (F, G) QRT-PCR analysis of *GATAβ4* and *SP1* mRNA in the fat body of wild type and transgenic female larvae on the 3 rd day of the fifth instar. (H) Morphology of external genitalia of transgenic and wild type moths. Scale bar, 500 μm. All experiments were conducted with three biological replicates, with each group consisting of five silkworms. Error bars represent mean ± SD (*n* = 3). Statistical significance was determined using two-tailed Student’s *t*-tests and is indicated by *P < 0.05, **P < 0.01, and ***P < 0.001.

It has been reported that RNAi knockdown of GATAa in *Aedes aegypti* and GATAβ4 in *Bombyx mori* significantly downregulated the expression of vitellogenin [47,48], suggesting that GATA family transcription factors may be involved in the regulation of sex-specific genes. Our previous study demonstrated that GATAβ4 directly binds to the *SP1* promoter, thereby enhancing the expression of SPs in *Bombyx mori* [49]. To investigate whether the GATAβ4 transcription factor is involved in the sex-specific regulation of *SP1* in the silkworm, a transgenic vector of GATAβ4 driven by the *LP3* promoter and dsRed marker gene under the control of the *3 × P3* promoter was constructed (Fig 4A), and transgene-positive individuals were obtained (S2A Fig). QRT-PCR showed that *GATAβ4* mRNA was overexpressed more than 100 times compared to wild type in males (Fig 4B), and *SP1* mRNA remarkably increased in overexpressing GATAβ4 male larvae (Fig 4C). Western blot results confirmed significant upregulation of GATAβ4 and SP1 proteins (Fig 4D and 4E). However, *SP1* mRNA expression was significantly downregulated with overexpressed GATAβ4 in female larvae (Fig 4F and 4G). We found that silkworm larvae overexpressing GATAβ4 were smaller than wild type and some exhibited an inability to pupate (S2B Fig). Furthermore, both male and female moths overexpressing GATAβ4 exhibited genital abnormalities (Fig 4H) and the expression of *dsxM* and *dsxF* was significantly downregulated (S3A and S3B Fig). These results suggest that GATAβ4 affects not only the expression and accumulation of SPs but is also involved in sexual differentiation.

### The interaction between GATAβ4 and doublesex affected the GATAβ4 binding to DNA

**Fig 5:**
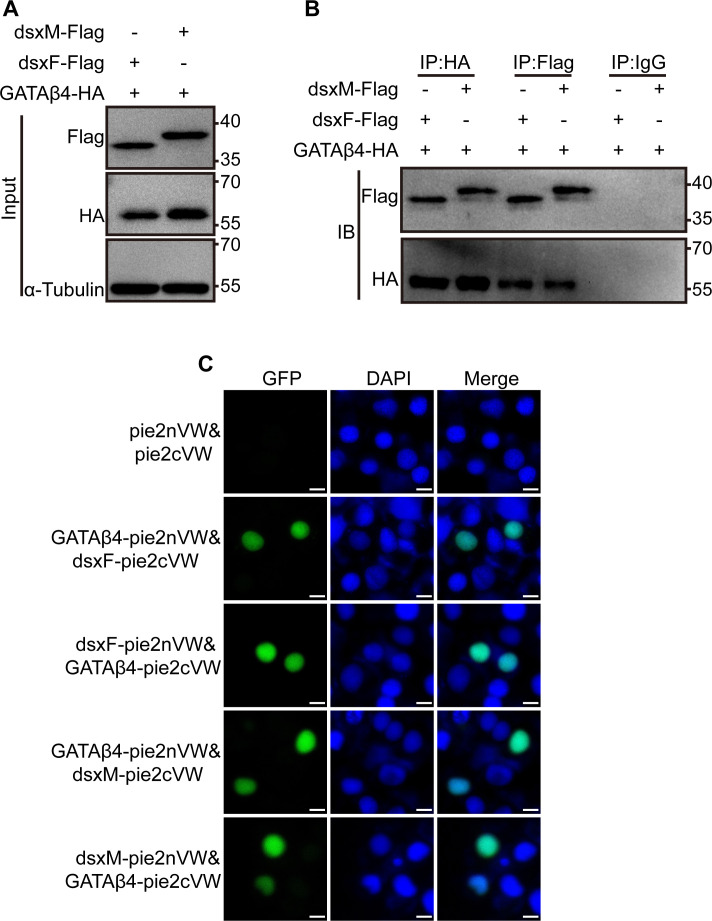
Interaction between doublesex and GATAβ4. (A, B) Co-immunoprecipitation (Co-IP) assay demonstrating interactions between GATAβ4 and dsxF or dsxM. The interactions of GATAβ4 and dsxF or dsxM were detected by bidirectional co-immunoprecipitation using anti-HA and immunoblotting with the reciprocal anti-Flag antibody or anti-Flag antibody and immunoblotting with the reciprocal anti-HA antibody. (C) Bimolecular fluorescence complementation (BiFC) assay showing interactions between GATAβ4 and dsxF or dsxM. BiFC assay was performed by co-transfecting GATAβ4-pie2nVW and dsxF-pie2cVW, dsxF-pie2nVW and GATAβ4-pie2cVW, GATAβ4-pie2nVW and dsxM-pie2cVW, dsxM-pie2nVW and GATAβ4-pie2cVW into BmE cells for 48 hours. Pie2nVW and pie2cVW were co-transfected as a control. Scale bar, 10 μm.

To investigate the relationship between GATAβ4 and doublesex, Co-immunoprecipitation (Co-IP) assays were performed to measure their interactions. The results showed that Flag-dsxF and Flag-dsxM were present in the anti-HA-GATAβ4 precipitates. Similarly, HA-GATAβ4 was detected in the anti-Flag-dsxF and anti-Flag-dsxM precipitates (Fig 5A and 5B). Bimolecular fluorescence complementation (BiFC) assays were also conducted to visualize the interactions of GATAβ4 with dsxF and dsxM in BmE cells. The results showed green fluorescence in cells co-expressing GATAβ4 and dsxF or dsxM, while no fluorescence was observed in control cells (Fig 5C). These results suggest that GATAβ4 interacts with either dsxF or dsxM.

**Fig 6:**
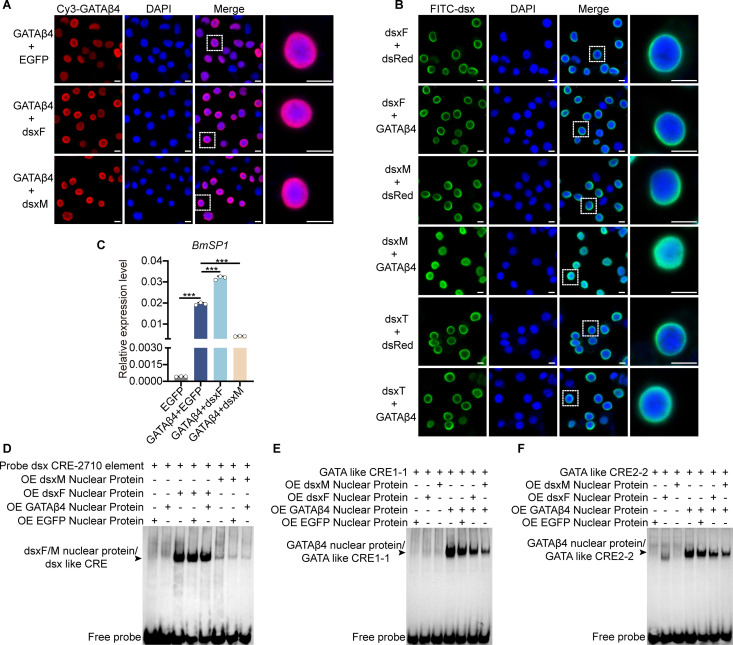
GATAβ4 induced dsxM into the nucleus and dsxM inhibited the ability of GATAβ4 to bind to DNA. (A) Subcellular localization of GATAβ4 in BmE cells after co-transfection with dsxF or dsxM, detected by immunofluorescence. Co-transfection with GATAβ4 and EGFP served as a control. (B) Subcellular localization of dsxF, dsxM or the C-terminal truncation of the dsxM (dsxT) in BmE cells after co-transfection with GATAβ4, detected by immunofluorescence. Co-transfection with dsxF, dsxM or dsxT and dsRed served as controls. (C) QRT-PCR analysis of *SP1* mRNA levels in BmE cells after co-transfection of GATAβ4 with dsxF or dsxM for 48 hours. All experiments were conducted with three biological replicates. Error bars represent mean ± SD (*n* = 3). Statistical significance was determined using two-tailed Student’s *t*-tests and is indicated by *P < 0.05, **P < 0.01, and ***P < 0.001. (D) Effect of GATAβ4 on the ability of dsxF or dsxM to bind DNA, assessed by electrophoretic mobility shift assay (EMSA) using biotin-labeled probes for the dsx CRE -2710 bp of the *SP1* promoter and nucleoproteins. (E, F) Effect of dsxF and dsxM on the ability of GATAβ4 to bind DNA, assessed by EMSA using biotin-labeled probes for GATA-like CRE 1–1 or GATA-like CRE 2–2 of the *SP1* promoter and nucleoproteins. BmE cells overexpressing dsxF or dsxM and GATAβ4 nuclear proteins were incubated for 20 minutes *in vitro* for EMSA. Controls included BmE cells overexpressing dsxF or dsxM and EGFP nuclear proteins. “+” indicated the presence of the sample in the lane; “-” indicated its absence. The black arrows point to the shifted bands. OE: overexpression. Scale bar, 10 μm.

We also investigated whether they affect each other’s subcellular localization. The results showed that dsxF and dsxM did not affect the localization of GATAβ4 in the nucleus (Fig 6A). DsxF, dsxM and the C-terminal truncation of the dsxM (dsxT) were located in the nuclear membrane. Interestingly, GATAβ4 drove dsxM to fully enter the nucleus but did not drive dsxF and dsxT (Fig 6B). This result suggested that the C-terminal region of dsxM is essential for its induction into the nucleus by GATAβ4. Next, we measured *SP1* mRNA levels after co-transfection of GATAβ4 with either dsxF or dsxM in BmE cells. The results showed that dsxF promoted the GATAβ4-induced upregulation of *SP1* mRNA expression, whereas dsxM inhibited this upregulation (Fig 6C). EMSAs were also performed to determine whether GATAβ4, dsxF, and dsxM affect each other’s DNA binding. The doublesex binding site upstream of the *SP1* promoter’s CRE -2710 bp was selected to assess whether GATAβ4 influences doublesex’s binding affinity to the *SP1* promoter. GATAβ4 directly binds to the GATA-like CRE 1–1 and CRE 2–2 elements in *SP1* promoter [49]. These two sites were used to evaluate whether doublesex influences GATAβ4’s binding to the *SP1* promoter. The results showed that GATAβ4 did not affect dsxF or dsxM binding to the CRE -2710 bp of the *SP1* promoter (Fig 6D). However, both dsxF and dsxM inhibited GATAβ4 binding to the GATA like CRE 1–1 and CRE 2–2 of the *SP1* promoter, with dsxM exhibiting a stronger inhibitory effect (Fig 6E and 6F). These results demonstrate that doublesex and GATAβ4 interact to regulate the female-specific expression of SP1 in *Bombyx mori*.

## Discussion

Previous studies have used a transgenic TALEN system to mutate dsxF in the silkworm, resulting in females lacking oocyte storage and exhibiting abnormal external genitalia [32]. Using transgenic TALEN and CRISPR/Cas9 systems to mutate dsxM or the common region of doublesex in the silkworm results in abnormal internal and external genitalia in both males and females [33]. These studies confirm that doublesex regulates the normal development of male and female genitalia in the silkworm. However, it remains unclear whether mutations in doublesex affect other dimorphic traits or which specific genes are impacted. In this study, we utilized the transgenic CRISPR/Cas9 system to mutate the common region of doublesex in the silkworm. The F1 progeny exhibited severe genital damage, preventing adults from mating and thus making it impossible to obtain doublesex mutant homozygotes or assess whether *doublesex* gene expression was reduced. Genomic testing of the F1 individuals revealed a 100% editing efficiency. Besides genital abnormalities, we observed a black spot at the last abdominal segment during the pupal stage, which might be due to internal genital malformations. Furthermore, we noticed that the wing pattern coloration in female moths became darker, although the molecular mechanism behind this phenomenon was not investigated. We focused on detecting the sex-specific gene *SP1* and observed a dramatic increase in males and a significant decrease in SP1 expression in females. In doublesex mutant females, SP1 did not decrease to the low levels observed in wild-type males. This is consistent with previous findings that overexpressing dsxF in male silkworm results in an upregulation of SP1, but did not reach the high levels seen in wild-type females [50]. These results suggest that the high expression of SP1 in female silkworms is not solely driven by dsxF. We speculate that additional positive regulatory factors may be required to assist dsxF in upregulating SP1 in females.

Although transgenic overexpression of GATAβ4 in female silkworms leads to reduced SP1 expression, our previous study showed that transient overexpression of GATAβ4 in female silkworm on the first day of the fifth instar using an insect baculovirus system significantly upregulated SP expression [49]. GATAβ4 continuous expression driven by the transgenic system used in this study may have influenced additional regulatory pathways. We observed that the transgenic overexpression of GATAβ4 affected the development and mating behavior of silkworm, and the expression of dsxM and dsxF was significantly downregulated (S3A and S3B Fig), which likely weakened the feminization process. These factors may contribute to the downregulation of SP1 expression. We speculate that this effect stems from a disrupted transcriptional balance. In female silkworms, SP1 is normally highly expressed, and transgenic overexpression of GATAβ4 may have disturbed this regulatory balance, thereby suppressing SP1 expression. In contrast, SP1 expression is minimal in males, allowing GATAβ4 overexpression to substantially enhance SP levels.

The yolk protein genes promoters, *yp1* and *yp2*, in *Drosophila melanogaster,* contain the FBE enhancer, which includes the aef1, dsxA, and bzip1 elements [51]. Both dsxF and dsxM proteins bind to dsxA. It is hypothesized that dsxF does not affect the binding of other transcription factors to bzip1, whereas dsxM interferes with this binding [29,30]. Additionally, the protein binding to the bzip1 site in the fat body may not be C/EBP, as C/EBP is expressed at very low levels in the fat body of *Drosophila melanogaster* [52]. The authors hypothesize that another family member, or members, is responsible for activation through bzip1, potentially binding more tightly to this site than DmC/EBP [29,30]. Both dsxF and dsxM proteins can bind to the promoter of the *vitellogenin* in *Bombyx mori* [50]. However, differential regulation of *vitellogenin* by BmdsxF and BmdsxM has not been reported. Here, we propose new hypotheses on how doublesex regulates genes, based on the differences in the ability of dsxM and dsxF to bind to the promoters of target genes. We hypothesize that the expression of *SP1* and *yolk protein* genes may share similar regulatory patterns, as GATAβ4 not only promotes SP1 expression but also contributes to the regulation of vitellogenin [49,53]. However, our current findings suggest that dsxM does not directly bind to the *SP1* promoter but instead interacts with GATAβ4. Moreover, there are obvious differences in the expression periods of yolk protein and SP1. Therefore, the sex-specific regulatory mechanism of vitellogenin warrants further investigation.

While investigating the interaction between doublesex and GATAβ4, we discovered an unexpected finding: both dsxF and dsxM localize to the nuclear membrane, a phenomenon not documented in prior studies. Previous research has indicated that dsxM and the masc gene co-localize within the nuclei of testicular cells in *Bombyx mori*, but the nuclear localization of dsxF in female silkworms has not been reported [54]. In another study, researchers identifying the doublesex gene and its function in *Artemia franciscana* found a potential nuclear localization signal (NLS) in the N-terminus of AfrDsxM, but not in AfrDsxF. They speculated that AfrDsxF might be unable to enter the nucleus, although this requires further direct evidence [55]. GATAβ4 can induce the nuclear translocation of dsxM but has no effect on the localization of dsxF. It remains unclear whether dsxF functions by localizing to the nuclear membrane or requires additional factors to translocate into the nucleus and exert its regulatory role. We favor the latter possibility, as previous studies have shown that the intersex (IX) protein interacts with dsxF and acts as a transcriptional cofactor to facilitate dsxF binding to DNA [56]. Additionally, we found that the binding of doublesex to GATAβ4 affects its binding to DNA, particularly dsxM. We hypothesize that this interaction interferes with the promotion of downstream gene expression by GATAβ4, thereby contributing to the sex-specific differential expression of SPs.

**Fig 7:**
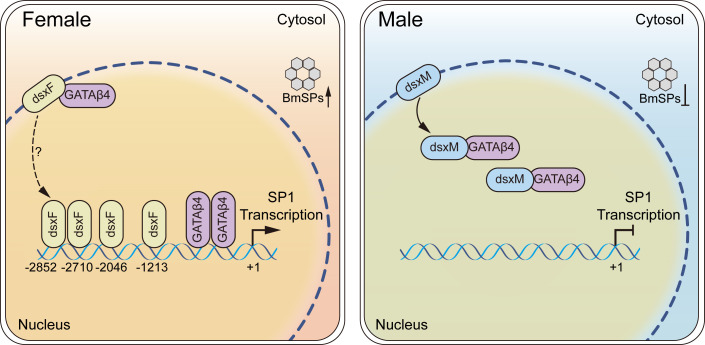
Schematic diagram illustrating the synergistic regulation of SP1 female-specific expression by doublesex and GATAβ4 in *Bombyx mori.* In female silkworms, both dsxF and GATAβ4 directly regulate the high expression of SP1. In male silkworms, dsxM exerts a weak direct effect on SP1 regulation. Instead, dsxM interacts with GATAβ4 to inhibit its binding to the *SP1* promoter, thereby preventing GATAβ4-mediated upregulation of SP1 and resulting in low SP1 expression.

In summary, our findings demonstrate that sex-specific pathways are essential for regulating sex-specific genes and that insect nutrition-related transcription factor GATAβ4 plays a significant role. Together with our earlier findings [49], this study reveals that both dsxF and GATAβ4 are directly involved in the regulation of high SP1 expression in female silkworms. In male silkworms, dsxM interacts with GATAβ4, altering its DNA-binding ability and thereby suppressing SP1 expression (Fig 7). Despite our many discoveries, this process remains complex and may involve additional transcription factors. The mechanism of sex differentiation may be investigated through the prediction of cis-regulatory elements in the promoters of downstream target genes and analysis of interactions among transcription factors. This study offers new insights into the regulation of sex-specific gene expression, highlighting the importance and complexity of controlling sexual dimorphism and sex-specific genes.

## Materials and methods

### Insects and cells

The *Bombyx mori* strain D9L was provided by the Academy for Advanced Interdisciplinary Studies Southwest University (Chongqing, China). Silkworm larvae were cultivated on fresh mulberry leaves in an incubator maintained at 25 °C and 75% relative humidity, with alternating 12 hours periods of light and darkness. The fat body was extracted from fifth instar silkworm larvae for total RNA or protein extraction. BmE cells were grown at 27 °C in Grace’s insect cell culture medium, which was enhanced with 10% (v/v) fetal bovine serum (Gibco, Invitrogen, USA).

### CRISPR/Cas9-mediated mutation

The binary transgenic CRISPR/Cas9 system was used to establish doublesex common (dsxC) region mutant silkworms. A unique single guide RNA (sgRNA) targeting the dsxC exon was designed with the CCtop online (https://cctop.cos.uni-heidelberg.de:8043/) [57]. The designed and synthesized single guide (sgRNA) fragment (listed in S1 Table) was used to construct a pBac [*3 × P3*-EGFP-*U6*-*dsxC* sgRNA] plasmid. This plasmid was mixed with a *piggyBac* helper plasmid (encoding piggyBac transposase) ensuring that both individual and mixed plasmid concentrations were no less than 500 ng/μL. The mixed plasmids were microinjected into embryos laid within 2 hours by the D9L strain, using a microinjector (Eppendorf FetoJet-4i, Germany) The injected embryos (G0) were reared to obtain G1 embryos. Positive sgRNA transgenic embryos from the G1 were screened with an enhanced green fluorescent protein (EGFP) using a fluorescent stereomicroscope (Olympus MVX10, Japan). The positive G1 individuals that expressed dsxC sgRNA were crossed with the nos-Cas9 transgenic line [45] (stored in our laboratory) to generate F1 heterozygous mutants.

### Vector construction and transfection

To analyze the *SP1* promoter activity, we amplified the *SP1* promoter sequence from the silkworm genome and created seven distinct truncated fragments. These fragments, which either included or excluded dsx CREs, were inserted into the pGL3-_basic vector (Promega, USA). The resulting recombinant vectors were designated based on the dsx CREs as pGL3-SP1-P2902, pGL3-SP1-P2819, pGL3-SP1-P2730, pGL3-SP1-P2677, pGL3-SP1-P2074, pGL3-SP1-P2013, and pGL3-SP1-P1233.

The *dsxF* (accession number: NM_001043406.1) and *dsxM* (accession number: NM_001111345.1) open reading frames were amplified from the cDNA of wild type fat body on the third day of the fifth instar and inserted into the psl1180-Hr3-OpIE-2 3 × flag SV40 vector (stored in our laboratory) for BmE cell overexpression. These vectors underwent recombination using the homologous recombination technique from the pEASY-Basic Seamless Cloning and Assembly Kit (Transgen, China). S1 Table contains the primers used for vector recombination.

In the transcription factor overexpression experiment, BmE cells cultured in 6-well plates were transfected with 2 μg of the overexpression plasmid alone. For the promoter activity assay, BmE cells in 24-well plates were co-transfected with 0.5 μg luciferase reporter plasmid, 0.5 μg overexpression plasmid, and 0.05 μg internal control reporter plasmid (pRL-VgP78M). The luciferase reporter and overexpression plasmids were mixed at a 1:1 ratio, and the luciferase reporter to internal control plasmid ratio was maintained at 1:0.1. All transfections were conducted using X-tremeGENE HP DNA transfection reagent (Roche, Basel, Switzerland) at a constant plasmid-to-reagent ratio of 1:2. After 48 hours of incubation, cells were collected for RNA extraction or promoter activity analysis.

### Quantitative real-time PCR (qRT-PCR)

Using TRLzol reagent (Invitrogen, USA), total RNA was extracted from the fat body of fifth instar (wild type, dsxC mutant, or transgenic overexpressed GATAβ4) or BmE cells. The PrimeScript RT Reagent Kit with gDNA Eraser (TaKaRa, Japan) was employed for cDNA reverse transcription, following the manufacturer’s instructions. Quantitative real-time PCR was conducted using the SYBR qPCR SuperMix Plus Kit (novoprotein, China). The qRT-PCR protocol consisted of 95 °C for 30 seconds, followed by 40 cycles of 95 °C for 3 seconds and 60 °C for 30 seconds. The *B. mori translation initiation factor 4A* (*BmeIF4A*) served as an internal control gene [58]. Primer Premier 5.0 software was utilized to design the primers (listed in S1 Table) for qRT-PCR.

### Protein extraction and western blot

Total protein was extracted from the fat body of fifth instar silkworms (wild type, dsxC mutant, or transgenic GATAβ4 overexpressed) using a RIPA lysis buffer containing 1% cocktail (Beyotime Biotechnology, China). The mixture was agitated at 4 °C for 30 minutes, then centrifuged at 12000 rpm for 30 minutes at 4 °C. The resulting supernatant, containing total protein, was either stored at -80 °C or immediately used after concentration measurement with a Bradford assay kit (Beyotime Biotechnology, China). For western blot analysis, 10 μg or 20 μg of protein samples were combined with 5 × loading buffer and separated using 12% SDS-PAGE. The proteins were then transferred to PVDF membranes, which were subsequently incubated with α-tubulin antibody (Beyotime Biotechnology, China), BmSPs antibodies, or BmGATAβ4 antibody (stored in our laboratory). The membranes were then treated with anti-rabbit/mouse IgG secondary antibodies (Beyotime Biotechnology, China). All antibodies were diluted 1:10000 in TBST containing 1% (w/v) skim milk powder. Imaging of the membranes was performed using a chemiluminescence gel imaging system (SINSAGE ChampChemi 610 Plus, China), while grayscale value calculations were conducted using ImageJ software [59].

### Immunofluorescence histochemistry

On the third day of the fifth instar, fat body from wild type and dsxC mutant silkworms were extracted in 1 × PBS and subsequently treated with 4% (v/v) paraformaldehyde for 30 minutes at room temperature. The samples were then washed three times in PBST buffer (1 × PBS containing 0.3% Triton X-100), with each wash lasting 10 minutes. Following this, the specimens were perforated using 0.5% Triton X-100 (Solarbio, China) for 15 minutes at room temperature and then blocked for 2 hours at room temperature using a mixture of 10% sheep serum albumin and 1% BSA in PBST buffer. The samples were then exposed to primary rabbit anti-SP1 antibody (1:500) overnight at 4 °C. After three 10 minutes washes with PBST buffer, the samples were incubated with secondary FITC Goat Anti-Rabbit IgG (H + L) antibody (1:500, Beyotime Biotechnology, China) for 1.5 hours. Finally, the specimens were washed three more times with PBST, each wash lasting 10 minutes.

BmE cells were seeded in 24 well plates containing climbing slides (Thermo, USA) and incubated overnight. Subsequently, the following combination plasmids were co-transfected into BmE cells, 0.5μg of each plasmid, and each well cells were co-transfected with a total of 1μg of the two plasmids. Each combination was tested in triplicate. The co-transfected plasmid pairs included: 1180-HA-GATAβ4 and 1180-EGFP-Flag, 1180-HA-GATAβ4 and 1180-dsxF-Flag, 1180-HA-GATAβ4 and 1180-dsxM-Flag. 1180-dsxF-Flag and 1180-dsRed, 1180-dsxF-Flag and 1180-HA-GATAβ4, 1180-dsxM-Flag and 1180-dsRed, 1180-dsxM-Flag and 1180-HA-GATAβ4, 1180-dsxT-Flag and 1180-dsRed, 1180-dsxT-Flag and 1180-HA-GATAβ4. After 48 hours of co-transfection, cells were washed twice with PBS and fixed in 4% (v/v) paraformaldehyde for 15 minutes at room temperature. The cells were washed three times with PBS and incubated for 10 minutes. The cells were permeabilized with 0.3% Triton X-100 (Solarbio, China) for 10 minutes at room temperature, followed by three 10 minutes PBS washes. The cells were blocked with 10% sheep serum albumin and 1% bovine serum albumin (BSA) in PBS for 2 hours at room temperature. The cells were incubated with primary mouse anti-HA tag antibody or mouse anti-Flag tag antibody (both from Beyotime Biotechnology, China) for 2 hours at room temperature. Three 10 minutes PBS washes were performed, followed by incubation with secondary Cy3 Goat Anti-Mouse IgG (H + L) or FITC Goat Anti-Mouse IgG (H + L) antibody (both from Beyotime Biotechnology, China) for 1.5 hours. The cells were washed three times with PBS, each wash lasting 10 minutes. Dilute both the primary and secondary antibodies 1:500 in 1 × PBS containing 1% BSA.

The fat body and cell samples were treated with 4’, 6-Diamidino-2-phenylindole (DAPI) (Beyotime Biotechnology, China) to stain the nuclei. This process was conducted at room temperature for 10–15 minutes. Subsequently, the samples were washed three times with PBS, each wash lasting 10 minutes. To prepare for microscopy, the climbing slide was inverted onto a glass slide containing an anti-fluorescence quenching agent. The slide was then sealed and examined using a confocal microscope (Zeiss LSM880, Germany).

### Electrophoretic mobility shift assay (EMSA)

Probes for dsx CRE -2882, -2852, -2710, -2046, and -1213 were created (detailed in S1 Table). These dsx CREs were predicted possible binding sites on the *SP1* promoter according to the website http://www.genomatix.de/ and https://jaspar.elixir.no/. These probes, with biotin-labeled 5’-termini (produced by Sangon Biotech), underwent annealing through a 10 minutes heating process at 95 °C, followed by gradual cooling to 25 °C. BmE cells were transfected with dsxF-Flag, dsxM-Flag, HA-GATAβ4, and EGFP overexpression plasmids for 48 hours. Subsequently, nuclear proteins were extracted using a Nuclear Protein Extraction Kit (Beyotime Biotechnology, China), adhering to the manufacturer’s protocol. The extracted nuclear proteins were then incubated with the biotin-labeled probes. The incubation process followed the guidelines provided in the EMSA/Gel-Shift Kit (Beyotime Biotechnology, China). To evaluate how dsxF, dsxM, and GATAβ4 might influence each other’s ability to bind DNA, nuclear proteins overexpressing these genes were combined for 20 minutes. They were also mixed with nuclear proteins overexpressing EGFP as controls. Subsequently, these mixtures were incubated to their respective probes for analysis. The resulting mixtures were separated using 6% (w/v) native polyacrylamide gels and underwent electrophoresis in TBE buffer (45 mM Tris-borate, 1 mM ethylenediaminetetraacetic acid, pH 8.3). Post-electrophoresis, the samples were transferred onto a nylon membrane (Roche, Indianapolis, IN, USA). The bands were then visualized using a chemiluminescence gel imaging system (SINSAGE ChampChemi 610 Plus, China).

### GATAβ4 overexpression in *Bombyx mori*

The open reading frames fragment of the *GATAβ4* (accession number: XM_038015622) was amplified by reverse transcription polymerase chain reaction (RT-PCR) and *LP3* promoter was amplified from the silkworm genome by RT-PCR. The *LP3* promoter and *GATAβ4* gene were assembled to create into *pSL1180* plasmid (stored in our laboratory). Then the recombinant *pSL1180* plasmid and *piggyBac* [3 × P3-dsRed] plasmid (stored in our laboratory) was recombined by homologous recombination method with pEASY-Basic Seamless Cloning and Assembly Kit (Transgen, China). The recombinant *piggyBac* [3 × P3-dsRed, LP3-GATAβ4] was mixed with the *piggyBac* helper plasmid (encoding piggyBac transposase) ensuring that both individual and mixed plasmid concentrations were no less than 500 ng/μL. The mixed plasmids were microinjected into embryos laid by D9L strain for less than 2 hours using a microinjector (Eppendorf FetoJet-4i, Germany). The injected embryos (G0) were reared to obtain G1 embryos. Positive transgenic overexpressed GATAβ4 embryos from the G1 were screened with dsRed using a fluorescent stereomicroscope (Olympus MVX10, Japan).

### Co-immunoprecipitation (Co-IP)

The overexpression plasmids GATAβ4 fused with the HA-tag and dsxF fused with the Flag-tag, or GATAβ4 fused with the HA-tag and dsxM fused with the Flag-tag were co-transfected into BmE cells for 48 hours. These cells were lysed to extract protein for Co-IP using Protein G Magnetic Beads (Thermo, USA). HA-tag antibody or Flag-tag antibody was added to 50 μL of Protein G beads for 30 minutes at 4 °C. The beads-antibody were collected using a magnetic rack and washed twice with PBS, each time for 5 minutes. After washing, the beads-antibody were resuspended in conjugation buffer containing 5 mM BS3 (Thermo, USA) and incubated for 30 minutes at room temperature. To terminate the crosslinking reaction, 12.5 μL of quenching buffer (1.5 M Tris-HCl, pH 7.5) was added. The beads-antibody were washed twice with PBS, each time for 5 minutes, and then incubated with cell protein at 4 °C overnight. The complexes were washed three times with PBS and eluted with 100 μL 1 × SDS loading buffer for 10 minutes at 95 °C. The eluates were analyzed by western blotting.

### Bimolecular fluorescence complementation (BiFC)

The GATAβ4, dsxF, and dsxM were inserted into the pENTR11 vector (Invitrogen, USA) to obtain the plasmids pENTR-GATAβ4, pENTR-dsxF, and pENTR-dsxM, respectively. These plasmids were then recombined into destination vectors (pie2nVW and pie2cVW) using Gateway reactions (Invitrogen, USA) to yield the plasmids GATAβ4-pie2nVW, GATAβ4-pie2cVW, dsxF-pie2nVW, dsxF-pie2cVW, dsxM-pie2nVW, and dsxM-pie2cVW. BiFC assays were performed by co-transfecting GATAβ4-pie2nVW and dsxF-pie2cVW, GATAβ4-pie2nVW and dsxM-pie2cVW, dsxF-pie2nVW and GATAβ4-pie2cVW or dsxM-pie2nVW and GATAβ4-pie2cVW into BmE cells. Pie2nVW and pie2cVW were co-transfected into BmE cells as a control. After 48 hours, these cells were stained with DAPI (Beyotime Biotechnology, China) for 10 minutes at room temperature. Fluorescence was observed using a confocal microscope (Zeiss LSM880, Germany).

### Morphological observation

External genital morphology of wild type, dsxC mutants, transgene overexpressed silkworm moths were observed and captured using a Stereomicroscope (Zeiss Stemi 2000-C, Germany). Wild type and dsxC mutants of silkworm pupae and moths were captured using a digital camera.

### Statistical analysis

The data from qRT-PCR, western blotting, and dual luciferase reporter assays were processed by GraphPad Prism software and transformed into histograms. All experiments were conducted with three biological replicates, with each group consisting of five silkworms. The data were shown as the mean ± standard deviation (*n* = 3). Student’s *t*-tests were used in GraphPad Prism software to calculate significant differences between treatments. According to the knowledge of biostatistics, P < 0.05 indicates statistical difference, marked with *, P < 0.01 indicates significant statistical difference, marked with **, P < 0.001 indicates extremely significant statistical difference, and marked with ***.

## Supporting information

S1 Table The primers used in this study. (XLSX)

S1 Data The original data and picture that were used to produce the figures in this study. (PDF)

S1 File The promoter sequences used in this study. (DOCX)

S1 Fig Fluorescence observation and verification of dsxC sgRNA and Cas9 expression in transgenic *Bombyx mori* lines. (A) Fluorescence microscopy of dsxC sgRNA G1 generation embryos and adults. White triangles indicated positive signals. (B) Amplification and sequencing of sgRNA from the G1 generation of the dsxC sgRNA silkworm strain. (C) Fluorescence microscopy of somites of Cas9 silkworm adults. dsxC sgRNA: dsx common region sgRNA silkworm strain; WT: wild type. Scale bar, 1 mm.
(TIF)

S2 Fig Transgenic overexpression of GATAβ4 in silkworms and phenotypic observations. (A) Fluorescence microscopy of first filial generation embryos and moths overexpressing GATAβ4, with white triangles indicating positive signals. (B) Phenotypic comparison of larvae to pupae stages between wild type (WT) silkworms and silkworms overexpressing GATAβ4 (LP3 > GATAβ4) driven by the *LP3* promoter. Scale bar, 2 mm.
(TIF)

S3 Fig Expression of dsxM and dsxF in silkworm transgenic overexpression of GATAβ4. (A) QRT-PCR analysis of *dsxM* mRNA in the fat body of wild type and transgenic overexpression GATAβ4 male larvae on the 3 rd day of the fifth instar. (B) QRT-PCR analysis of *dsxF* mRNA in the fat body of wild type and transgenic overexpression GATAβ4 female larvae on the 3 rd day of the fifth instar. All experiments were conducted with three biological replicates, with each group consisting of five silkworms. Error bars represent mean ± SD (*n* = 3). Statistical significance was determined using two-tailed Student’s *t*-tests and is indicated by *P < 0.05, **P < 0.01, and ***P < 0.001.
(TIF)
