# Supplementary material for: Doublesex and GATAβ4 synergistically regulate the sex-dimorphic expression of storage protein 1 in Bombyx mori
Source: PLoS Genet. 2025 Jul 11;21(7):e1011762. doi: 10.1371/journal.pgen.1011762 (PMC12250667; doi:10.1371/journal.pgen.1011762)
Supplement: S1 Data — (PDF) [file pgen.1011762.s002.pdf]

Fig 2A

	WT-M			dsxC-M			
BmSP1	0.02893	0.028053	0.030449	11.9406	13.85819	12.24755	P<0.0001
BmSP2	80.48545	80.52871	72.08018	126.9476	123.2882	120.3498	P=0.0002
BmSP3	38.06721	32.59225	35.20636	69.66565	70.30523	66.91703	P<0.0001

Fig 2B

	WT-F			dsxC-F			
BmSP1	54.4712	45.23157	50.13708	19.35861	19.3427	20.72458	P=0.0004
BmSP2	191.9876	180.0484	175.8845	168.2385	149.632	151.6378	P=0.0265
BmSP3	133.7385	121.5759	123.6536	93.3786	94.00618	80.70105	P=0.003

Fig 2D

α-Tubulin	BmSP1	BmSP2/SP3
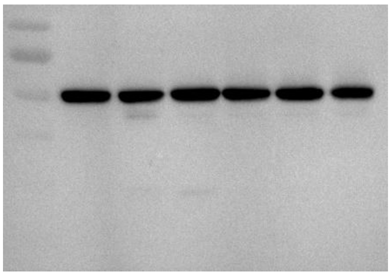	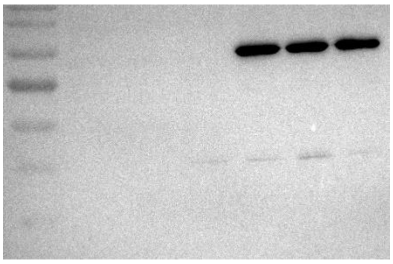	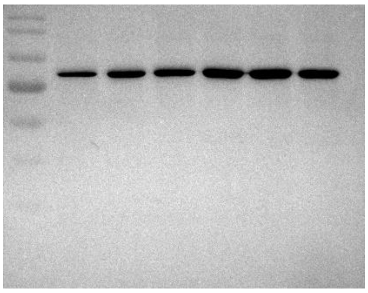

Fig 2E

BmSP1		BmSP2/SP3	
WT-M	dsxC-M	WT-M	dsxC-M
0.11115794	1.204197729	0.64502833	1.24211003
0.089333319	1.004298935	0.88762977	1.08020871
0.101224897	1.199576097	0.91490496	1.08085965
	P<0.0001		P=0.0347

Fig 2F

α-Tubulin	BmSP1	BmSP2/SP3

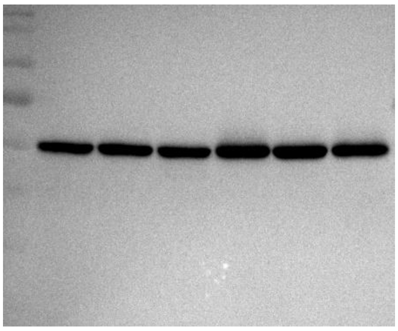 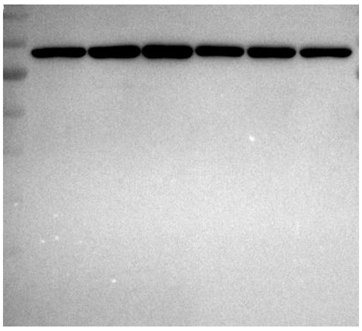 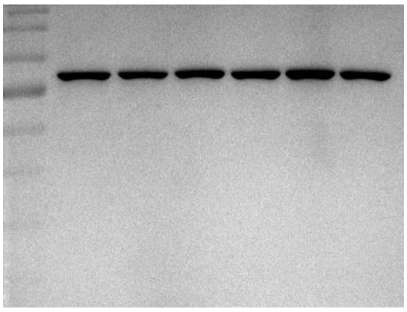

Fig 2G

BmSP1		BmSP2/SP3	
WT-F	dsxC-F	WT-F	dsxC-F
1.10083006	0.84588191	1.00303558	0.727407253
1.05147518	0.79641972	0.817451447	0.758732604
1.219501	0.76701589	0.911882364	0.717763411
	P=0.0043		P=0.0328

Fig 3A (BmSP1)

EGFP	dsxF	dsxM
0.000429	0.000682	0.000632
0.000413	0.000654	0.000638
0.000446	0.000725	0.000636
	P=0.0003	P<0.0001

Fig 3A (BmSP2)

EGFP	dsxF	dsxM
0.0000129	0.0000147	0.0000181
0.00000967	0.0000132	0.0000158
0.0000127	0.0000124	0.0000132
	P=0.2488	P=0.0885

Fig 3A (BmSP3)

EGFP	dsxF	dsxM
0.000182	0.000256	0.00024
0.000172	0.000241	0.000218
0.000169	0.000264	0.000229
	P=0.0005	P=0.0019

Fig 3C

	1180-EGFP			1180-dsxF			1180-dsxM		
2902	7.516129	7.571429	8.518519	29	24.125	23.94737	14.30556	15.12121	18.76667
2819	9.054054	9.129032	10	21.97143	24.48571	23.72222	12.02703	12.57895	11.83784

2730	10.82857	11.67568	10.57895	30	27.60606	24.2	18.77143	18.075	17.5122
2677	14.12903	16.06061	11.625	32.42857	36	37.59375	18.9697	19.97297	15.15
2074	11.81818	9.540541	9.514286	48.69444	46.81579	45.4359	25.58824	23.05714	21.25
2013	7.567568	5.294118	6.966667	33.09091	31.0303	35.62857	12.69048	13.94118	15.31429
1233	7.516129	7.571429	8.518519	40.21622	42.43243	38.91429	17.13889	22	21.14286

Fig 4B

WT-M	GATAβ4-M
0.001076	0.172665
0.00095	0.170539
0.000956	0.184833
	P<0.0001

Fig 4C

WT-M	GATAβ4-M
0.003894	16.33142
0.003864	18.71292
0.004095	16.36832
	P<0.0001

Fig 4D

α-Tubulin	GATAβ4	BmSP1	α-Tubulin	GATAβ4	BmSP1
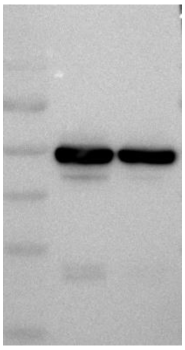	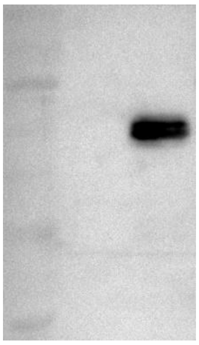	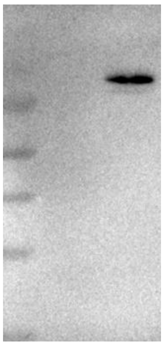	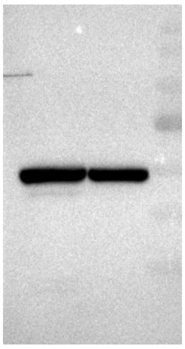	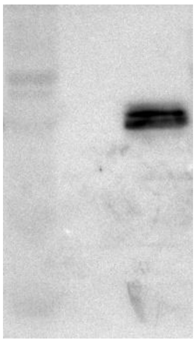	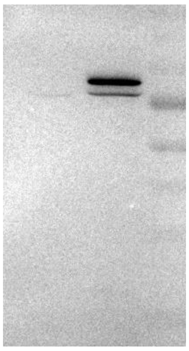
α-Tubulin	GATAβ4	BmSP1			

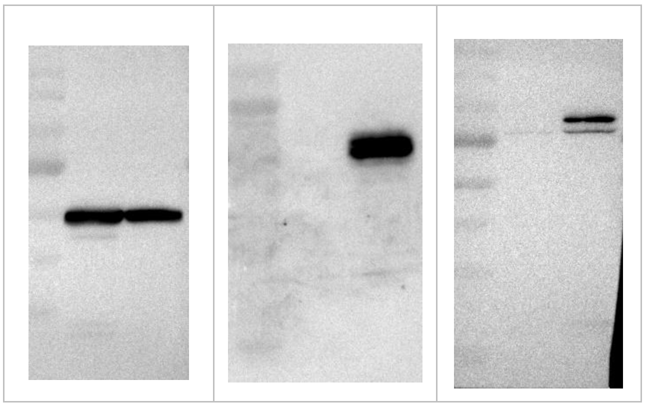

Fig 4E

GATAβ4		BmSP1	
WT-M	GATAβ4-M	WT-M	GATAβ4-M
0.042796014	1.037880496	0.011052696	0.566422911
0.05877952	1.077040252	0.007049677	0.871607877
0.065578844	1.198627098	0.012494785	0.720773576
	P<0.0001		P=0.0013

Fig 4F

WT-F	GATAβ4-F
0.001889	0.013386
0.001958	0.013274
0.001914	0.012915
	P<0.0001

Fig 4G

WT-F	GATAβ4-F
31.23003	8.372185
29.88886	12.19462
30.71365	11.46333
	P<0.0001

Fig 5A

Anti-Tubulin	Anti-HA	Anti-Falg

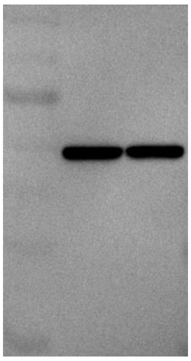	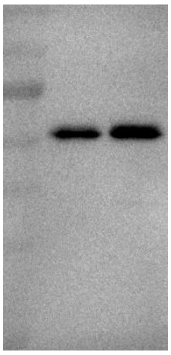	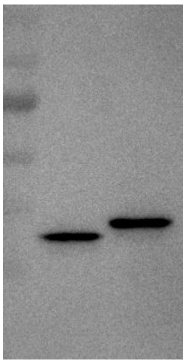

Fig 5B

Anti-HA	Anti-Flag
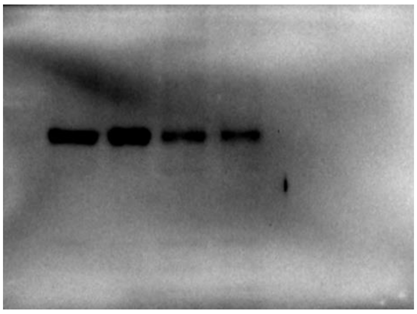	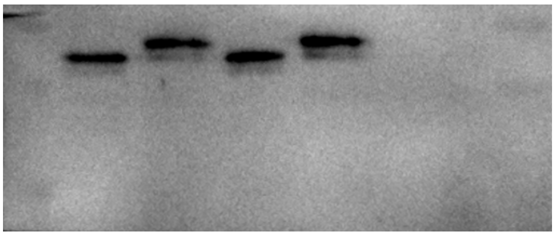

Fig 6C

EGFP	EGFP+GATAβ4	GATAβ4+dsxF	GATAβ4+dsxM
0.000429	0.020235	0.03095	0.004142
0.000413	0.019478	0.032101	0.004243
0.000446	0.019082	0.032595	0.004429
	P<0.0001	P<0.0001	P<0.0001

Fig S3A

	WT-M			GATAβ4-M			
dsxF	0.0000591	0.0000505	0.0000629	0.0000258	0.0000422	0.0000336	P=0.0169
dsxM	0.003612	0.004143	0.00427	0.001989	0.001821	0.002385	P=0.0018

Fig S3B

	WT-F			GATAβ4-F			
dsxF	0.002382	0.00297	0.002505	0.001161	0.00127	0.00112	P=0.0015
dsxM	0.0000356	0.0000171	0.00000171	0.0000603	0.0000202	0.0000202	P=0.4045
