# Supplementary material for: Doublesex and GATAβ4 synergistically regulate the sex-dimorphic expression of storage protein 1 in Bombyx mori
Source: PLoS Genet. 2025 Jul 11;21(7):e1011762. doi: 10.1371/journal.pgen.1011762 (PMC12250667; doi:10.1371/journal.pgen.1011762)
Supplement: S1 File — (DOCX) [file pgen.1011762.s003.docx]

>Sequence of *3 × P3* promoter

TAATTCGAGCTCGCCCGGGGATCTAATTCAATTAGAGACTAATTCAATTAGAGCTAATTCAATTAGGATCCAAGCTTATCGATTTCGAACCCTCGACCGCCGGAGTATAAATAGAGGCGCTTCGTCTACGGAGCGACAATTCAATTCAAACAAGCAAAGTGAACACGTCGCTAAGCGAAAGCTAAGCAAATAAACAAGCGCAGCTGAACAAGCTAAACAATCGGGGTACCGCTAGAGTCGACGGTACCGCGGGCCCGGGATCCACCGGTCGCCACC

>Sequence of *immediate-early 1* (*IE1*) promoter

ATTTGCAGTTCGGGACATAAATGTTTAAATATATCAATGTCTTTGTGATGCGCGCGACATTTTTGTAAGTTATTAATAAAATGCACTGACACGTTGCCCGACATTATCATTAAATCCTTGGCGTAGAATTTGTCGGGTCCGTTGTCCGTGTGCGCTAGCATGCCCGTAACGGACCTTGTGCTTTTGGCTTCAAAGGTTTTGCGCACAGACAAAATGTGCCACACTTGCAGCTCTGCTTGTGTGCGCGTTACCACAAATCCCAACGGCGCAGTGTACTTGTTGTATGTAAATAAATCTCGATAAAGGCGCGGCGCGCGAATGCAGCTGATCACGTACGCTCCTCGTGTCCCGTTCAAGGACGGTGTTATCGACCTCAGATTAATATTTATCGGCCGACTGTTTTCGTATCCGCTCACCAAACGTGTTTTTGCATTAACATTGTATGTCGGCGGATGTTCTGTATCTAATTTGAATAAATAAATGATAACCGCATTGGTTTTAGAGGGCATAATAAAAAAAATATTATTATCGTGTTCGCCATTAGGGCAGTATAAATTGACGTTCATGTTGAATATTGTTTCAGTTGCAAGTTGACATTGGCGGCGACACGATCGTGAACAACCAAACGAC

>Sequence of *nanos* (*Nos*) promoter

GGTTGTAATGTAGGTATTGGTACAAATATTATATTTGTACTTAACGCGTTATACCTATTGATTGGGTCAAGCGCGATATGCGAGACTTCTTTTTTATTTTATTTAGATGGGTGGACGAGCTCACAGCCCACCTGGTGTTAAGCAGCTATCGGAGCCCATAGACACCTACAACGTAAATGAGCCACCAACCTTGACATATAAGTTCTAAAGTCTCAACAAGGTCTCAAGTATAGTTACAACGGCTGCCCCACCCTTCAAACCGAAACCCATTACTGCTTCACGGCAGAAATAGGCAGGGTGGTGGTAGGTACCTACCCGTGCAGACTCACAAGAGGTGAACAAAATAAAAGACAAATATATAGCAAATACATAAATAGTTTTTTTCGATATTTTAGGGTTATAATATTAATTTCTGGCGTTTCAAATACTTTCGTTTTCGTGGTCTATTGTTTACTAAAGCACGTGTGGCGTCATCTAGCGTTGAGTAGTACAAACAGGATTACACGTGCCAAAATTGTCGGTAGGTACCTTTGAAGCTGAAGCCGAAAAGTCGGCGCCTTTTTAAGTTAAAGACAAAAAGGAACCGCCCTTTGAATGCGTTTGCCGAAAGTTTGTATATTTTTAATGAACTCAAAACACAAGCATTCGATCCATCGTGGTGTGCTGACGTGCAACCCAAATCGTGATTACATTTAATAAAAGGTAAGAAACAAATTAAATAAGCACACCTACTTAATGAGAAATTTCGCTAATTTTATAATTATGAATTAAATTAATAGTTTTTATAATAATTAGCTTCAAATTTAAAAGTAAATAACAACACGTTGATTTTTTCTTAATGTGACTGCACAGTGGGAAGGCGTTCCAAGGCGAAGCTAACTATATTATGTATTATAGCTGCTATTATGTGTTAAGTGCTATATATTGATAGATAATGTTCGCGGGATGCCTACAATCACTACCAAGTTAATGCCAGTGATTTCTATTTATCAAATCGTATAGAACATATTTCCAGTTTCCTATACTATGGCTTAATGAAGCTTATACAATCATAGTCTTATTTCAACCTTTTTAAATTACGCCTTACCATCCAGTAGTCTAGTGCCGGACTTAGCTCATTCCCATAAATCCTATCTAAATCTATATTTAGTTGTTATTAAATTAGCATAGATTAATACACTATTTAGTAAAATCGTTTTCATGTGAGATTAGTCCCTCATTGCTAATTGCGTTTCAGGATTTTTTAACAATCATAAGTTAAACTGCTAGCCACCGCGTATTTATTAACCATATAAATAATGCAAAAAAGTAGCACTGGCTAGTCAGATAATAGCATGAATATGTTAGGTATTTTCTAAATATACTACAGTAGGTACTAGATTTTTGTTAAATTCCACTGAATAAAATTGTTTCAATTATTGTGTTAATAAAAAAGTCTGTTCAACCACTAGTTTTAACACTACTCCTTAATTTTGATTAAATTTTGTCATGAACGCGTAAATGTTCAAGACATAATATTTATTTATGGTATTAGATACCTATCGTTAATGGCGTCCATAAAATTAATATTGAGCTGTATTTTAATGATGGTATCAGTATAATGATTATTTAAATCGGCACTGAATTTTTTTAGTATTTTTTTGGTAGTAAGTACTTTTCTTACACATAAATTGTTGATTTTTTTGAGTCTATCGTCTCATATGTATAAAAAGTTGATTCAAAATAAGAATGCTTCCGGGTGCTATCGGATGTTCGGTCATTAGAATATAATTCCTAATAATATTCTACTTCATTAAAGTCATTAAAATTTAAAGTGCCTAAATTTTTGTTTAAAGTACATACGTAGTAAAAACGTAAATGAATGTGTAAGTCATGTTTTTATTATGTACATTACTTATCTTTTCAGATAAAATAATAAAACA

>Sequence of *U6* promoter

AGCTGTCCAAGGAATGCGTAGCAGCTTTCTCCAGCAATACATTTCAAACGCCTCAATCTTTTTGCGTTCCTTTTTCCTGAGACACCAAGTCTCCTAAAGTCATGATGATTGACCTAAAAGAATCAATACAGTTTAATAAATTTATAAGTATTAGGTTATGTAGTACACATTGTTGTAAATCACTGAATTGTTTTAGATGATTTTAACAATTAGTACTTATTAATATTAAATAAGTACATACCTTGAGAATTTAAAAATCGTCAACTATAAGCCATACGAATTTAAGCTTGGTACTTGGCTTATAGATAAGGACAGAATAAGAATTGTTAACGTGTAAGACAAGGTCAGATAGTCATAGTGATTTTGTCAAAGTAATAACAGATGGCGCTGTACAAACCATAACTGTTTTCATTTGTTTTTATGGATTTTATTACAAATTCTAAAGGTTTTATTGTTATTATTTAATTTCGTTTTAATTATATTATATATCTTTAATAGAATATGTTAAGAGTTTTTGCTCTTTTTGAATAATCTTTGTAAAGTCGAGTGTTGTTGTAAATCACGCTTTCAATAGTTTAGTTTTTTTAGGTATATATACAAAATATCGTGCTCTACAAGTGATG

>Sequence of *LP3* promoter

AGTATAGTTACAACGGCTGCCCCACCCTTCAAACCGAAACGCATTACTGCTTCACGGCAGAAATAGGCAGGGAGGTGGTATCTAACCGTGCGAATCCATACGAAAATATTAATATTAGCAAAACAAATATACCTTTCACATGGCATGTTTTAAACTACCACGAACAATGTGAATTTTTAAATGTGTCCATTAAAATTACACATTTAAATTATAATGTTGACGGCTTGATAATTTCACTCAATCAATAATAAACTATATCTTTATTTCAATGCACTTTCATTTGACATTTGAACTATGATGTTGATATTGCATCTGACGTTTTTTAATTCAAAACAATTTCGAGTATAAAAGGCAGAGTTTCAAAGGAAACAGGCAGTTCGTTCTTGGGTAACACACAGGTGAGATACATTTTGTTTTTAACTCTGGAGAATCCGTTTCGGATACCCAGTCGTGGGGGGTAACAGACCGGGTTATGTCAGACTTCGGTTCCTCCAAGGAAGAGGGAGACCGAGGTCCTCCTCTTCTTCTAATTCCTGTCGAGAGTCTACGTCTTGAGATATCTACCTACCACACAAAAACGTTTTCTTCTATTTAGCGTTTGTTAAATTGTAAGAGTTTGAGAAACCAATTGGCCGATATTTCGACCTCTGGCATTTTTTTCATCACTCCGCTGACTTTTCTTATTCTTTTTATTGCTTAGATGGGTGAACGAGCTCACAGCCCACCTGGTGTTAAGTGGTTACCGGAGCCCATAGACATTTACAACGTAAATGCCCCACCCACCTTGAAATTTAAGGTCTAAGATCTCAAGTATAGGTACTTCTTGTACTGGTCTCCAAACACCGATCGTATGAATTTTGTATCAGTGGATATAAAATTATACACTAAGATGTTTATGTGTCTAAGCTTTCAAAGAAGTCAAATATATAATATACTTTTTTATTTAACAATTAATTTGTCAAGTTCGTTTTTGCTATATACTCACAAAATCTGCGACCGTTTTGTCTCATATATACATCAAATATACATATTATGTTCAATTCTCAATGTGTATAATTCAACTTACGTTTTTAAAATTCTAATCCTTAACAAATAATTTTACATATTGCAGGACTCGACTCGAC

>Sequence of *OpIE-2* promoter

CATGATGATAAACAATGTATGGTGCCAATGTTGCTTCAACAACAATTCTGTTGAACTGTGTTTTCATGTTTGCCAACAAGCACCTTTATACTCGGTGGCCTCCCCACCACCAACTTTTTTGCACTGCAAAAAAACACGCTTTTGCACGCGGGCCCATACATAGTACAAACTCTACGTTTCGTAGACTATTTTACATAAATAGTCTACACCGTTGTATACGCTCCAAATACACTACCACACATTGAACCTTTTTGCAGTGCAAAAAAGTACGTGTCGGCAGTCACGTAGGCCGGCCTTATCGGGTCGCGTCCTGTCACGTACGAATCACATTATCGGACCGGACGAGTGTTGTCTTATCGTGACAGGACGCCAGCTTCCTGTGTTGCTAACCGCAGCCGGACGCAACTCCTTATCGGAACAGGACGCGCCTCCATATCAGCCGCGCGTTATCTCATGCGCGTGACCGGACACGAGGCGCCCGTCCCGCTTATCGCGCCTATAAATACAGCCCGCAACGATCTGGTAAACACAGTTGAACAGCATCTGTT

>Sequence of *SP1* promoter

TGCCTGTTGACTTTGGTGCCTACTTAACTCGAGGTGGGCCGCAATTCTTTAGTTGACGAAATAAAAGTCGTAAACAAATTTTAGCAGTCAATTTTACTTTGTATATTTAACCTTTCAAGATTATGACATTGTCATTATCGTGCTGGGTGATATAAACCTGGTTGTTATTTCTTTACAAAAAAAAAGTGCACGTAATTTGACGTTTAATGTCAAGCACATAAATATGAAAATCGCGTATCAATTCCACGCGACTAAAAACAAGTACTTACTTTGTCGTTTGCAATCGAGTTACACGGTTCAATGGTCGTTGTTATGTAAACAATATTAATCGCATTAATTATTTATTCTTATCTTGTTTGTTTTTGTAAGCTGGTGTCAAGCTAATGTATTGTATGTTTTATTCCGAGATTTTTTATCGCTCTACGTAGGCAGACTAACCCATGTGATGTCAACTGGTAATTGAAGCCAATTCACATCAGATGGGTTATGAATTGCGCCATCCACTTTGAGACCCATAGGTCTTGACAGGCCAATGTCTCAATTGTATTATATAACGGTAGCCAGCCCCCCAGTCGGAGCGGATACTTGCTTCGTGGCAGGAATAGGCACAACGATGGTGCTTACCTGCGCATGGAATATATCGGGCGATTTAATCCATACCACAACAGAAGATCACATGTCATTGCAACCGCATCTACGTACGTGTGACGCACGTGAAGGAATAACATCGTGTAATAAAAATTAAACCCGCAAAATTATAATTTGCGTAATTACTGGTGGTAGGACCTCTTGTGAGTCCGCGCGGGTGGGTACCCCCACCCCGCCTATTTCTGCCGTGAAGCAGAAATGCGTTTCGGTTTGAAGGGTGGGGCAGCCGTTGTAACTATACTTGAGACCTTAGAACTTATATCTCAAGGTGGGTGGCGCATTTACGTTGTAGATGTCTATGGGCTCCAGTAACCACTTAGCACCTGGTGGGCTGTGAGCTCGTCCATTCATATAAACAATAAAAAAAAATTTTTTGGTGGCAGATTCGAGAATCGACACCCTTGTCAGATTGTAAACCAATCTAGCATTTTCCCTGCAAGAATTCACAGCAGAATTTTTTGTTGACATTTTGTCGATTGATGTTATGACACAACTGCTCTTGTGCATGTTCACTGAACGTCATGATACAGGCTTCAACAAATGAGAATCAAAAAGAGTCTTAAGCGATAGACAAGAGCGAAATGAAAACGTTGCGATTCAAATAATTAAAGAATAAAATATACGCAATAAGGCAGAAAGATTTAACCTCTCTTAACCTACAATATAGTTAAAAACAGTGTAAGAATCCTAATTAATTCGCTTGTTTGCATGGTACGGGGCAGCTGAAGCTACTGGTGGCAACGTACAGGTAGCACCACACTGTCCATTTTAGCCGTAAGTCAGTCACGCGTCATGGTCGGTAGGGTAGAACGACGATGCGACTGTGCCGGAATTGAAATTTCGCAGGTGGGTACAAAATTTGGTAGAGAAGACCTTCCACCGAGCCGGTGGAACTGCTCGATGGACCGATAGTCCGAATTTAATTGTTAGGAATTTATTTAATGCAACGTATTGTGAAATTATCGTTAGCGCAATTCAGGCATCTGTCAAATATCTTGTGTTCTTCGAAGGAATACAATACAAAATAATATCGTGTAATAAAAGCCAAACCTGCAATAATTATAATTTACTGTTCAATTGGGACAGACTTCAAATCTCTAAGGGGTGGAGGCATTCACGTTGTAGTGTCTATAGGCTCCGGCAACCACTTAAGACCAGGTGAGCCTCGAGCAGTTCAATAGTCAACGCAATATATATACGAAAGTCTTTCATTGATTTTTTTACATCGGTACTCCACGCGAAGTTTACGCGAGATTTAGACGAGATTTACAAGATAAAAGACTAATGATTTCGGAAGTAAAAAATACTAATGAGAATATGAGTTTGAATCACATAAGTTTTTGATAGATTTGTCTTGTATATCAATGTTTATTATTTTATCACAAATGTTGCGCCGATGGACATGGCAATGGCAGGATTATTTTGCGGCAGGAACATGCAAGACAACCGAACACGTCACAAACAGAACCACAGCACGCCTCACTTCGAGTTTTTTTTATCGCGATCGACAATTAGACGACGTCGTGAGGGCCACGATAGAAATTTAAATGATTTATGTCAACTCAAAATCCATAATAAAAACTCTATGAGGGGTTCCTAAACAAACCTCAGTCCAGAAAAACGGTGAATTAAACCCAGCGGACTAATCTTGGAGCGACCCTCAACATTATAACATGCTGCCTGTAAATTAACCATTGAATTTATAATTTTCTTTAGTGCACAAGCGTTCCTTGATAGGTTTCTTAAATCGTATTGTAATGATTGTCGTACCCTTGAGATATAGTTATGACTGACGTATGACCGACATGATGCATTGATCATGGTAGTTGCACGACACGTCCTATCTCCAAAACTTTTGGCCCGAAAGTTCCCAAGTCCCCCGTATTCATTTTGTTACAGAGATTATTAAAAAAAGTGAATATTTCCATAAAGAATTTTGAATGGTAGAATCTCGTCTCAATAGATACTTCAAAAATCAATTGACAAATCGAGAAGAAATGGATACCTAACTGTTGTGATTTTGAAATTATTTATCAGTATTTATTGGTTCAAAAATCCGATTTTAAAAAGAAAAAAGTGACCGATCGATCTAGGCCGGCCTACACGTTACCGCGCCGGAAATATGGCCTATAATGCTATAAGTGCTAGTAAAATGAAAAAATCCATAAAAATTACAAAATCATCTCATCTCCGTGCATTAAAATCACAAAGAAGAGAGTTGATCCCTGGGGTTATCTGCTGTAACGAGAGCTGATAATGGTTGGATAGTAACGTGAAAACAAGTTTCACGTTACATATATATAGGCAGACGGAATGGTTTGTGTTC
