# Supplementary figures and images for: Doublesex and GATAβ4 synergistically regulate the sex-dimorphic expression of storage protein 1 in Bombyx mori
Source: PLoS Genet. 2025 Jul 11;21(7):e1011762. doi: 10.1371/journal.pgen.1011762 (PMC12250667; doi:10.1371/journal.pgen.1011762)

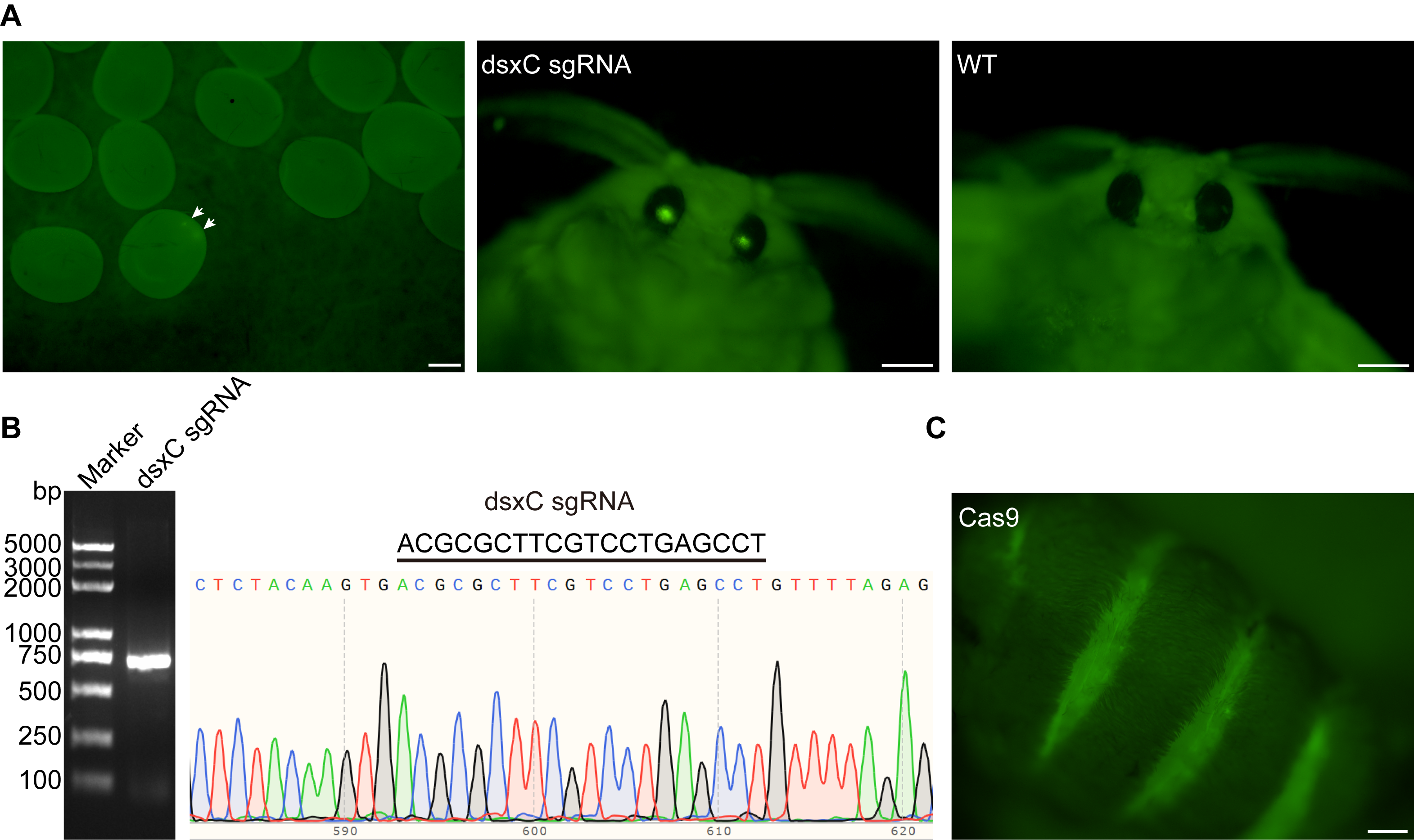

Supplement: S1 Fig — (A) Fluorescence microscopy of dsxC sgRNA G1 generation embryos and adults. White triangles indicated positive signals. (B) Amplification and sequencing of sgRNA from the G1 generation of the dsxC sgRNA silkworm strain. (C) Fluorescence microscopy of somites of Cas9 silkworm adults. dsxC sgRNA: dsx common region sgRNA silkworm strain; WT: wild type. Scale bar, 1 mm. (TIF) [file pgen.1011762.s004.tif]

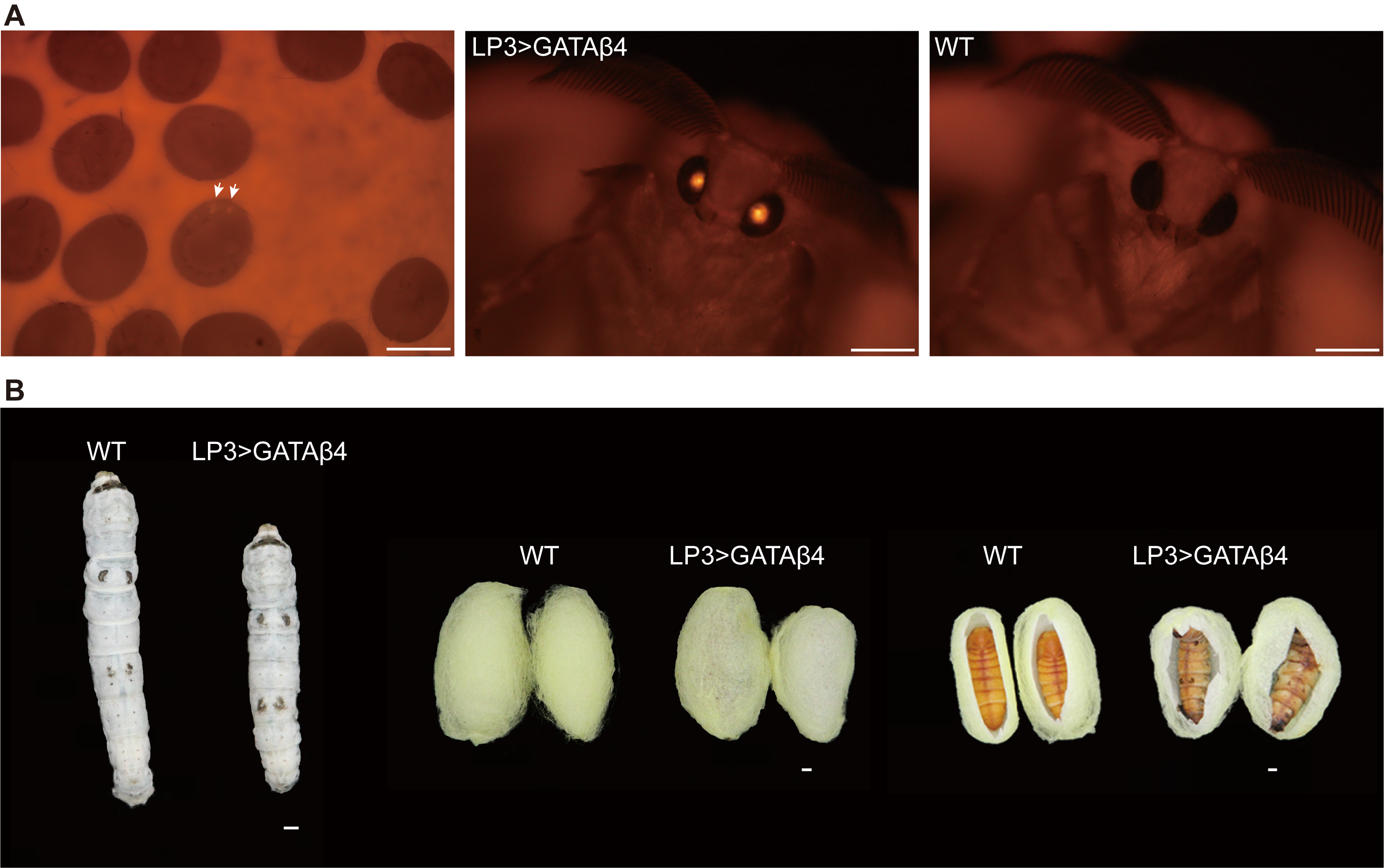

Supplement: S2 Fig — (A) Fluorescence microscopy of first filial generation embryos and moths overexpressing GATAβ4, with white triangles indicating positive signals. (B) Phenotypic comparison of larvae to pupae stages between wild type (WT) silkworms and silkworms overexpressing GATAβ4 (LP3 > GATAβ4) driven by the LP3 promoter. Scale bar, 2 mm. (TIF) [file pgen.1011762.s005.tif]

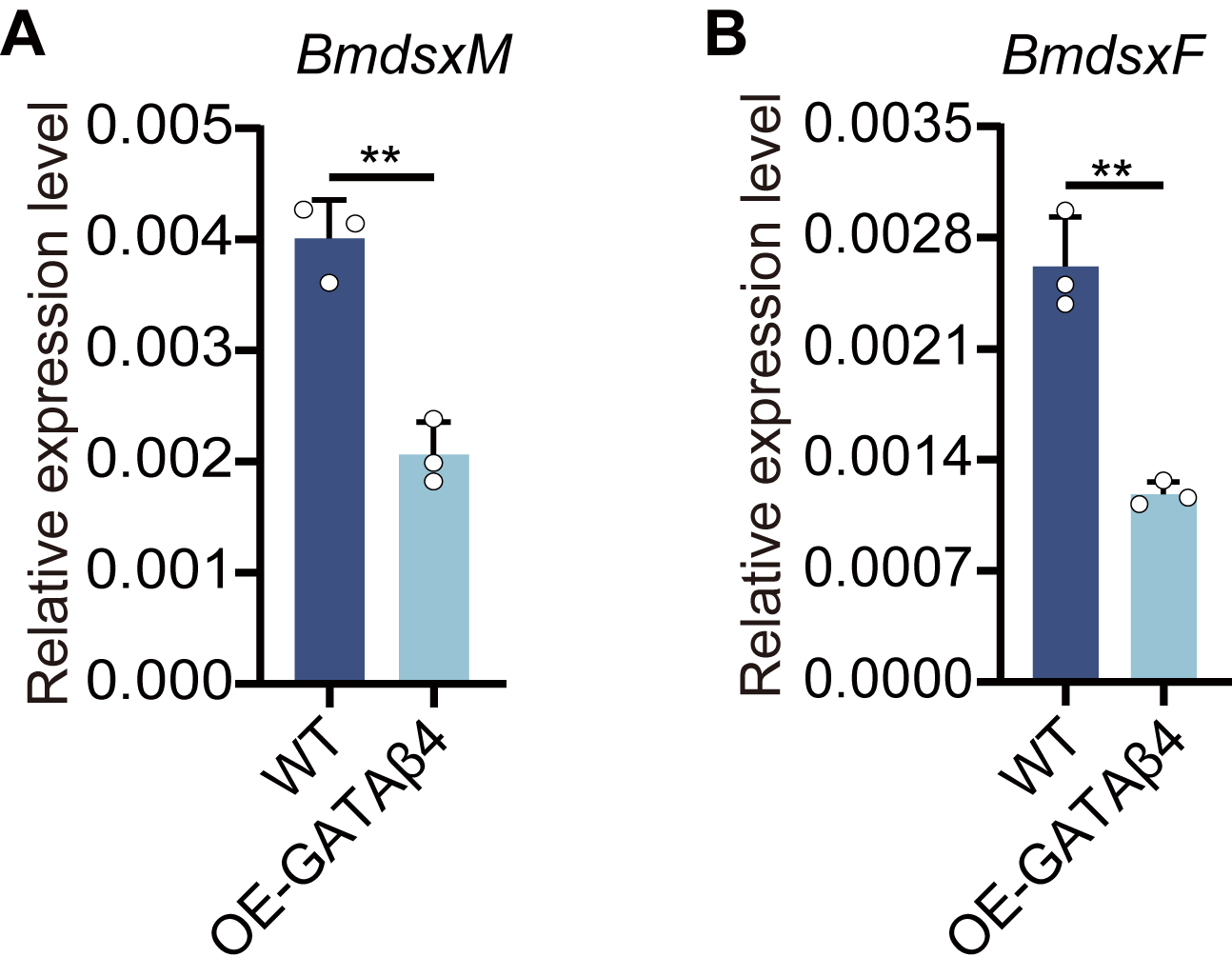

Supplement: S3 Fig — (A) QRT-PCR analysis of dsxM mRNA in the fat body of wild type and transgenic overexpression GATAβ4 male larvae on the 3 rd day of the fifth instar. (B) QRT-PCR analysis of dsxF mRNA in the fat body of wild type and transgenic overexpression GATAβ4 female larvae on the 3 rd day of the fifth instar. All experiments were conducted with three biological replicates, with each group consisting of five silkworms. Error bars represent mean ± SD (n = 3). Statistical significance was determined using two-tailed Student’s t-tests and is indicated by *P < 0.05, **P < 0.01, and ***P < 0.001. (TIF) [file pgen.1011762.s006.tif]
